# Culinary treatments impact the digestibility and protein quality of edible insects: a case study with *Tenebrio molitor* and *Gryllus assimilis*

**DOI:** 10.3389/fnut.2024.1399827

**Published:** 2024-05-31

**Authors:** Barbora Lampová, Ivo Doskočil, Martin Kulma, Michal Kurečka, Lenka Kouřimská

**Affiliations:** ^1^Department of Microbiology, Nutrition and Dietetics, Faculty of Agrobiology, Food and Natural Resources, Czech University of Life Sciences Prague, Prague, Czechia; ^2^Department of Zoology and Fisheries, Faculty of Agrobiology, Food and Natural Resources, Czech University of Life Sciences Prague, Prague, Czechia

**Keywords:** culinary treatment, nutrition, protein content, protein digestibility, DIAAS

## Abstract

The escalating global population is anticipated to intensify the demand for high-quality proteins, necessitating the exploration of alternative protein sources. Edible insects are a promising solution, owing to their nutritional richness and sustainability. However, their digestibility and protein quality, particularly after culinary treatment, remains underexplored. In the present study, we investigated the effects of various culinary treatments on the protein digestibility of two insect species, *Tenebrio molitor* and *Gryllus assimilis*. Our findings revealed that culinary treatments such as boiling, roasting, drying, and microwave heating significantly influenced the digestibility of both insect species. Notably, drying emerged as the most effective method, leading to a substantial increase in digestibility. Furthermore, we assessed protein quality using the digestible indispensable amino acid score (DIAAS) and found that the choice of the calculation method significantly influenced the evaluation of protein quality. By including the sum of the anhydrous amino acids, we eliminated the potential overestimation of protein content and obtained a more reliable assessment of protein quality. Our results underscore the importance of culinary treatments and calculation methods in determining the suitability of insects as protein sources for human nutrition.

## 1 Introduction

The world population is experiencing significant growth and is projected to reach 9 billion by 2050 (1). With an increasing population, there is an anticipated surge in the demand for food, particularly for high-quality proteins essential for optimal human metabolism. This poses a global challenge in ensuring an adequate food supply (2, 3). Consequently, it is imperative to explore new sources of high-quality protein suitable for human consumption. Edible insects are considered a possible solution (4, 5). In some regions of Africa and Asia, edible insects are traditional foods, whereas in Europe they remain an unconventional part of the diet for a limited number of people. In contrast to conventional livestock, edible insects have minimal space requirements, are feasible for vertical farming, produce significantly less greenhouse gases and ammonia than cattle or pigs, and certain insect species can be raised on organic waste (6). One of the major advantages of insect farming is its efficient feed conversion ratio, which indicates the amount of feed required to gain 1 kg of weight. This high efficiency is attributed, amongst other factors, to the poikilothermic nature of insects, as they do not have a stable body temperature, and thus do not expend energy on its maintenance. In this regard, insects outperform other livestock (7). The nutritional value of edible insects is influenced by the insect species, developmental stage, sex, environment, and other factors. Edible insects are rich sources of proteins, fats, vitamins, and minerals (8–10). The average crude protein content in edible insects ranges from 13 to 80 g/100 g of the dry matter (3, 11). These values surpass those of most plant-based protein sources, and at the upper limit, they may compete with certain animal-based sources (12).

Nutritional quality cannot be inferred solely from the protein or amino acid composition. Their biological availability, particularly of essential amino acids, is crucial. Biological availability or digestibility indicates the amount of protein available for absorption after passing through the upper part of the gastrointestinal tract (13–15). The issues of digestibility and the impact of culinary preparations on the nutritional value of insects have not been sufficiently explored (16). Edible insects are mostly consumed after culinary treatment, making this question of paramount importance (17, 18). Culinary treatment can improve the safety, quality, and taste characteristics of edible insects; however, it can also enhance or reduce the digestibility of proteins (19). Heat treatment can induce protein denaturation, unfold the polypeptide chain, make individual peptide bonds more accessible to digestive enzymes, and consequently increase protein digestibility (20). Digestibility can also be reduced by the formation of disulphide bonds between sulphur-containing amino acids via heat treatment and redox reactions and also by the formation of aggregated structures. In products rich in fat and protein, increased temperatures can lead to the formation of lipid-protein complexes, which are less susceptible to digestive enzymes, ultimately resulting in decreased digestibility. Also, lipids themselves are able to form micelles that surround proteins thus protecting them from enzymatic proteolysis, which reduces protein digestibility. Moreover, the type and composition of insects can also influence their digestibility (21, 22).

Therefore, in this study, we focused on the impact of culinary treatments such as boiling, roasting, drying, and microwave heating on the protein digestibility of two edible insect species, *Tenebrio molitor* and *Gryllus assimilis*. The selection of the model species was based on their popularity amongst consumers and availability on the market particularly in Europe, Asia, and South America (23, 24).

## 2 Materials and methods

### 2.1 Samples and sample treatment

Whilst crickets are usually consumed in full grown stages, adult mealworm beetles contain chinone-based repellent glands, which make them disgusting. Thus, this study investigated the species in stages, which are commonly consumed. The edible insects used in this Study *T. molitor* larvae and adult *G. assimilis*, were obtained from the Insectarium of the Czech University of Life Sciences, Prague. The insects were reared at a temperature of 27°C ± 2°C and a relative humidity of 40%–50%. *Gryllus assimilis* was fed chicken feed prepared at the university experimental facility, comprising wheat (from the Czech University of Life Sciences fields), soybean meal extract (Prowena s.r.o., Czech Republic), rapeseed oil (Fabio s.r.o., Czech Republic), and a vitamin and mineral premix containing calcium and monocalcium phosphate (Trow Nutrition Biofaktory s.r.o., Czech Republic). *Tenebrio molitor* was fed a mixture of oat bran and chicken feed (4:1). Both insects were starved for 24 h prior to harvesting to empty their gastrointestinal tracts. The insects were killed by blanching (300 g of the insect sample was placed in 3 L of boiling water for 20 s). The killed insects were subsequently subjected to different culinary treatments including boiling, roasting, drying, and microwave heating. Boiling involved keeping 300 g of insects in 3 L of boiling water for 30 min. For roasting, approximately 150–200 g of edible insects were placed on a preheated pan without oil and roasted for 5 min. Drying was performed in aluminium containers containing 60 g of *G. assimilis* or 80 g of *T*. *molitor* and the samples were dried in an oven (Memmert, Germany) at 80°C for 15 h. *Gryllus assimilis* samples (240 g) were heated in a microwave oven (Samsung GE83X, 800 W) for 1-min followed by a 4-min break. The total duration of the microwave heating was 8 min. *Tenebrio molitor* samples (280 g) were subjected to microwave heating using the same procedure, with a total heating time of 10 min. The culinarily treated samples were subsequently lyophilised (Trigon Plus lyophiliser, Czech Republic), homogenised (MultiDrive basic laboratory mill, IKA, Germany), and stored at −80°C until the analysis.

### 2.2 Nutritional analysis

Nutritional analyses were performed in triplicate. Dry matter content was determined by overnight drying in the oven at 103°C ± 2°C. Nitrogen content was determined using the Kjeldahl method (ISO 5983-1:2005) with a Kjeltec 2400 analyser (FOSS, Hillerød, Denmark). Crude protein content was calculated using a nitrogen-to-protein conversion factor of 6.25. Amino acid analysis was performed by Eurofins Food & Feed Testing, Czech Republic s.r.o., which is accredited by the Czech Institute for Accreditation (accreditation number: 1546). Tryptophan content was determined by liquid chromatography using a fluorescence detector, and other amino acids were determined by ion chromatography with ultraviolet detection.

### 2.3 Static *in vitro* digestion model

A simulated *in vitro* static digestion model was prepared according to the methodology outlined previously (25). In brief, 5 g of the sample was mixed with 5 mL of simulated salivary fluid (15.1 mM KCl; 3.7 mM KH_2_PO_4_; 13.6 mM NaHCO_3_; 0.15 mM MgCl_2_·6H_2_O; 0.06 mM (NH_4_)_2_CO_3_; 1.1 mM HCl; 1.5 mM CaCl_2_·2H_2_O), 0.5 mL amylase (75 U/mL in total digestate) and distilled water to achieve final ratio of 1:1 (vol/vol). The samples were incubated for 2 min at 37°C and a pH of 7.

The oral bolus was mixed with 8.1 mL of simulated gastric fluid (6.9 mM KCl; 0.9 mM KH_2_PO_4_; 25 mM NaHCO_3_; 47.2 mM NaCl; 0.12 mM MgCl_2_·6H_2_O; 0.5 mM (NH_4_)_2_CO_3_; 15.6 mM HCl; 0.15 mM CaCl_2_·2H_2_O), 1 mL pepsin (2,000 U/mL in total digestate), and gastric lipase (60 U/mL in total digestate). The pH was adjusted to 3 by adding 5 M HCl and the distilled water was added to achieve final ratio of 1:1 (vol/vol). The samples were incubated for 120 min at 37°C.

The gastric chyme was mixed with 11 mL of simulated intestinal fluid (6.8 mM KCl; 0.8 mM KH_2_PO_4_; 85 mM NaHCO_3_; 38.4 mM NaCl; 0.33 mM MgCl_2_·6H_2_O; 8.4 mM HCl; 0.6 mM CaCl_2_·2H_2_O), 2.5 mL bile (10 mM in total digestate), and 5 mL pancreatin (trypsin activity 100 U/mL in total digestate). The pH was adjusted to 7 by adding 1 M NaOH and the distilled water was added to achieve final ratio of 1:1 (vol/vol). The samples were incubated for 120 min at 37°C. Digestion was stopped by freezing the samples at −80°C.

### 2.4 Determination of total digestibility

The digested samples were centrifuged (at 3,500 × g for 10 min) to separate the undigested proteins from the amino acids (digested part). Total digestibility was subsequently determined by comparing the amino acid content of the undigested and digested samples according to Equation 1.

Equation 1: *In vitro* protein digestibility was calculated from the sum of anhydrous amino acids (AA) in digested and undigested samples.


(1) $$\mathrm{Total}\;\mathrm{digestibility}\;\left(\%\right)=\frac{\sum \mathrm{AA}\;\mathrm{in}\;\mathrm{digested}\;\mathrm{samples}}{\sum \mathrm{AA}\;\mathrm{in}\;\mathrm{undigested}\;\mathrm{samples}}*100$$

### 2.5 Digestible indispensable amino acid score determination

The digestible indispensable amino acid score (DIAAS) was calculated according to the Food and Agriculture Organization of the United Nations (FAO) methodology (26) which defines the DIAAS value as the lowest calculated value for the digestible indispensable amino acid reference ratio (DIAA) as a percentage. DIAA (Equation 2) was determined for each amino acid as the digestible indispensable amino acid (IAA) content in 1 g of food protein (in mg) divided by milligrammes of the same dietary indispensable amino acid in 1 g of reference protein (amino acid scoring pattern).

Equation 2: Calculation of digestible indispensable amino acid (DIAA).


(2) $$\mathrm{DIAA}=\frac{\mathrm{digestible}\;IAA\;\mathrm{content}\;\mathrm{in}\;1\;g\;\mathrm{protein}\;\mathrm{of}\;\mathrm{food}\;\left(\mathrm{mg}\right)}{\mathrm{the}\;\mathrm{same}\;IAA\;\mathrm{in}\;1\;g\;\mathrm{of}\;\mathrm{the}\;\mathrm{reference}\;\mathrm{protein}\;\left(\mathrm{mg}\right)}$$

Two methods were used to calculate the DIAAS values (Equation 3). The first method uses digestible IAA recalculated to 1 g of crude protein, calculated by multiplying the total nitrogen content by a conversion factor of 6.25 (TN × 6.25), whereas the second method uses the sum of individually determined amino acids in the dry matter (sum of anhydrous AA). The following amino acids were used to determine the sum of amino acids: alanine, arginine, aspartic acid, glutamic acid, glycine, histidine, hydroxyproline, isoleucine, leucine, lysine, ornithine, phenylalanine, proline, serine, threonine, tyrosine, valine, cysteine, cystine, methionine, and tryptophan.

Equation 3: Calculation of digestible indispensable amino acid score (DIAAS).


(3) $$\mathrm{DIAAS}=\mathrm{lowest}\;\mathrm{value}\;\mathrm{of}\;\mathrm{DIAA}\times 100\;\left(\%\right)$$

### 2.6 Statistical analysis

The statistical analysis of the data involved the application of two-way analysis of variance (ANOVA), followed by post-hoc analyses using Scheffe’s method at a significance level set at α = 0.05. Tested factors were ‘insect’ and ‘culinary treatment’. For results in Table 1, one-way ANOVA was used, followed by post-hoc analyses using Scheffe’s method at a significance level set at α = 0.05. The Statistica 13.2 software package (StatSoft, Inc., United States) was used for this purpose. *F*-values were calculated to determine the differences amongst the various groups. The results are presented as arithmetic means (x) with corresponding standard deviations based on observations from three independent samples.

**Table 1:** Total *in vitro* protein digestibility of *Tenebrio molitor* and *Gryllus assimilis*.

Culinary treatment	Control—no treatment	Boiled	Roasted	Dried	Microwaved
***Tenebrio molitor***					
Sum of AA in undigested samples (g/100 g DM)	53.16 ± 3.64^a^	50.44 ± 3.60^a^	52.33 ± 3.65^a^	50.01 ± 3.52^a^	53.11 ± 3.72^a^
Sum of AA (digested samples) (g/100 g DM)	45.70 ± 3.18^a^	44.02 ± 3.07^a^	48.76 ± 3.40^a^	47.37 ± 3.30^a^	45.03 ± 3.30^a^
Digestibility (%)	85.97 ± 4.84^a^	87.27 ± 4.73^a^	93.17 ± 4.98^a^	94.72 ± 4.83^a^	45.03 ± 4.97^a^
***Gryllus assimilis***					
Sum of AA in undigested samples (g/100 g)	61.64 ± 4.23^a^	60.06 ± 4.27^a^	60.63 ± 4.20^a^	64.50 ± 4.50^a^	59.92 ± 4.18^a^
Sum of AA in digested samples (g/100 g)	46.98 ± 3.29^a^	46.92 ± 3.28^a^	47.72 ± 3.34^a^	54.98 ± 3.82^a^	46.73 ± 3.26^a^
Digestibility (%)	76.22 ± 5.36^a^	78.13 ± 5.39^a^	78.71 ± 5.37^a^	85.23 ± 5.90^a^	77.98 ± 5.30^a^

AA, amino acids; DM, dry matter. Values are expressed as means ± standard deviations (*n* = 3), numbers on the same row followed by different lowercase letters are statistically different (*p* ≤ 0.05).

## 3 Results

Total *in vitro* protein digestibility, determined as the ratio of the total amount of amino acids in the dry matter of samples not subjected to the *in vitro* digestion model and digested samples, ranged from 76.2% to 94.7% for the analysed samples. Table 1 provides an overview of the sum of amino acids in the two insect species examined before and after *in vitro* digestion and their subsequently calculated digestibility.

The sum of all amino acids present in individual samples before *in vitro* digestion shows that *G. assimilis* is a richer source of proteins than *T. molitor*. *Tenebrio molitor* samples contained approximately 10 g/100 g more amino acids compared to *G. assimilis* samples. The highest amino acid content was found in samples processed by drying for both types of insects.

Insect species had the greatest influence on digestibility (*p* < 0.0001; *F* = 27.59). A lower but statistically significant effect was observed for culinary treatment (*p* < 0.037; *F* = 3.15). *Tenebrio molitor* exhibited higher *in vitro* digestibility than *G. assimilis*, regardless of the culinary treatment method. The highest digestibility values were achieved by drying, with *T. molitor* reaching 94.72%, and *G. assimilis* reaching 85.23%. Drying led to an 11% increase in digestibility compared with untreated culinary samples in both insect species. In both cases, the second most effective method was frying; however, for *T. molitor*, this increase was more pronounced than that for the untreated sample. An increase in digestibility was also observed in *T. molitor* samples treated by boiling. A slight decrease in digestibility was observed after microwave heating. For *G. assimilis* samples, all culinary treatments had a positive effect on protein digestibility.

**Figure 1:**
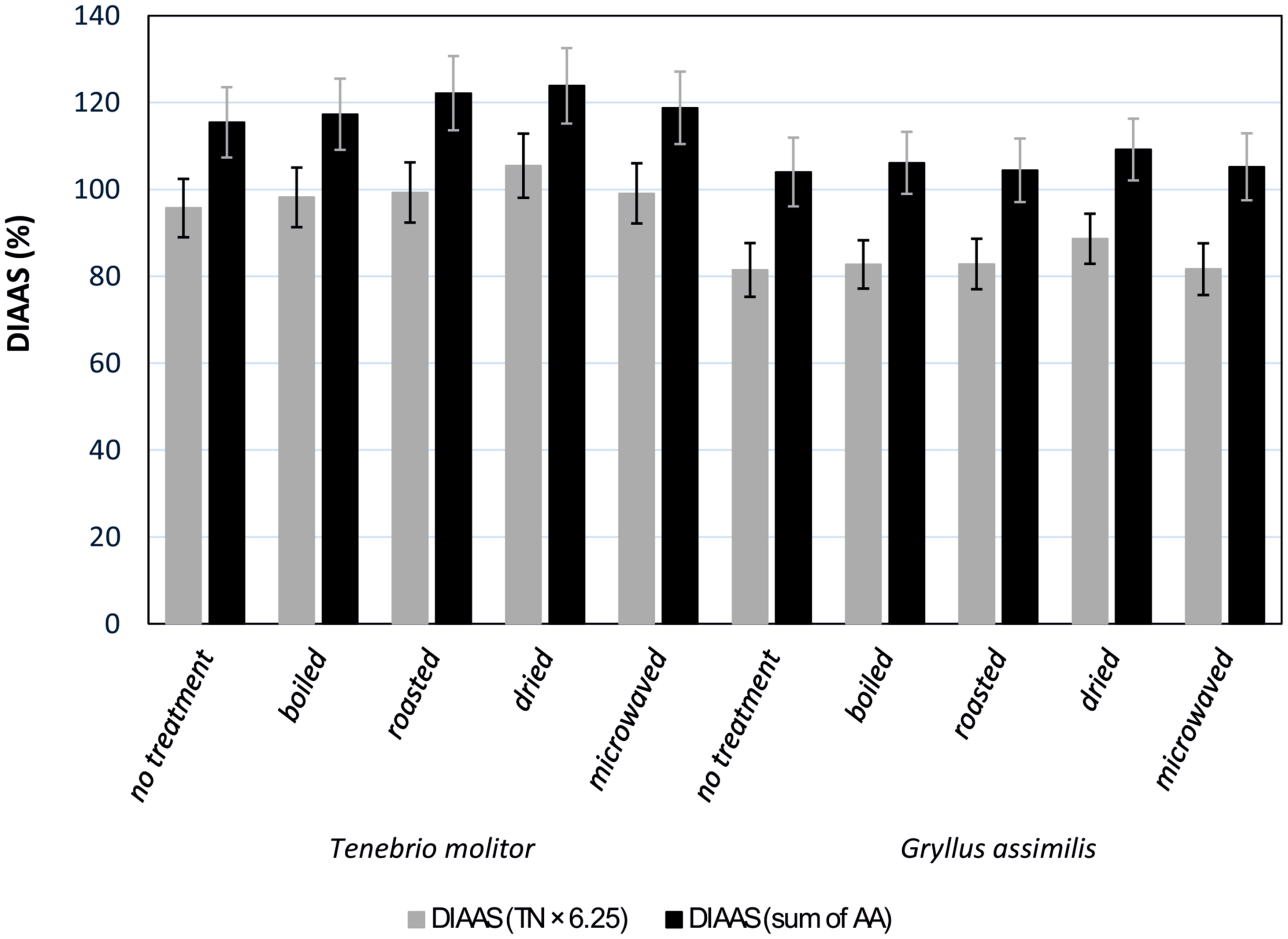
Digestible indispensable amino acid score (DIAAS) of *Tenebrio molitor* and *Gryllus assimilis* calculated using two methods. The first method involved recalculating the digestible indispensable amino acids to 1 g of crude protein, obtained by multiplying the total nitrogen by a conversion factor of 6.25 (TN × 6.25). The second method utilised the sum of individually determined amino acids in dry matter (the sum of anhydrous AA).

The DIAAS results (Supplementary Table S1) indicate that all culinary treatments influenced the protein digestibility of edible insects, both for *T. molitor* and *G. assimilis*. Overall, higher DIAAS values were obtained when calculating the sum of amino acids (sum of AA) rather than using the calculation based on crude protein obtained by multiplying the nitrogen content by a factor of 6.25 (TN × 6.25). In general, *T. molitor* exhibited higher DIAAS values. Untreated *T. molitor* reached a DIAAS of 95.72% if the DIAAS calculation utilised TN × 6.25, or 115.44% if the calculation utilised the sum of all determined amino acids in the dry matter. Moreover, this value further increased with various culinary treatment methods. Both insect species had the lowest DIAAS values in the untreated culinary samples and the highest values in the dried samples (Figure 1).

## 4 Discussion

Different approaches for assessing insect protein digestibility have been published. The first attempt was reported by Ramos-Elorduy et al. (11), who conducted an intensive study on insects collected freely from the Mexican countryside. In this study, the *in vitro* digestibility was approximately 85% for most of the investigated species. Approximately 20 years later, a similar study was conducted in Uganda, where the chemical composition and digestibility of three commonly consumed insect species (*Syntermes*, *Macrotermes*, and *Brachytrupes*) in the region were examined. The protein digestibility of these species, based on *in vitro* methods, ranged between 30% and 50% (27).

The literature indicates significant inconsistencies in digestibility evaluations, attributed to variations in the digestion method, the approach employed for assessing digestibility, and the species or developmental stage of the insect (17). Despite these differences, insect protein digestibility is generally considered high for most species, ranging from 80% to 90% (28). Similar digestibility values were found in our study, with *T. molitor* digestibility ranging from 84.79% to 94.72% and *G. assimilis* from 76.22% to 85.23%. The lower limits of *T. molitor* digestibility were comparable to soybeans (86%), oatmeal (86%), and rice (88%). However, it should be noted that in the case of *T. molitor*, the proteins present had a more favourable composition in terms of essential amino acid profiles than plant protein sources. At the upper end, *T. molitor* protein digestibility was comparable to that of fish and meat (94%) and milk and cheese (95%). However, eggs have an even higher amino acid digestibility (97%). *Gryllus assimilis* digestibility, which was approximately 10% lower than *T. molitor*, was comparable to that of plant protein sources (29).

However, the main objective of our work was not only to monitor the digestibility of edible insects but also to analyse the effect of culinary treatment on the protein digestibility of this potential food source. The issues of digestibility and the impact of kitchen treatments on the nutritional value of insects have been insufficiently explored in the literature (16). Similar to conventional animal products, as the consumption of raw insects is uncommon, knowledge of the effects of culinary treatments on digestibility is essential (30).

In our study, an increase in digestibility was observed with all culinary treatments, except for microwave heating of *T. molitor*. Increased digestibility after culinary treatment was also confirmed by Caparros Megido et al. (19) who examined the effect of culinary treatments on the protein digestibility of *T. molitor*. In this study, the digestibility of culinary untreated *T. molitor* was found to be 85% ± 2.5%, which is consistent with our findings. Culinary treatments such as vacuum cooking, frying, boiling, or oven cooking led to an increase in digestibility by 2.2%–6.5%. This increase can be explained by the exposure to denaturing temperatures, which unfold polypeptide chains, making the protein more accessible to digestive enzymes (28). Conversely, Mancini et al. (31), who studied the effects of various culinary treatments (oven cooking at 70°C for 30 min, oven cooking at 150°C for 10 min, deep frying, pan frying, microwaving, vacuum-boiling, and steaming) on *T*. *molitor* larvae, found that all processes except for oven cooking at 70°C for 30 min led to a reduction in protein digestibility compared with non-processed larvae (31). This different trend compared with our study could be due to high temperatures, which may have led to amino acid oxidation, formation of oxidative products, interactions between proteins and aldehyde products of lipid oxidation, or protein aggregation (32).

In a study by Manditsera et al. (17), a reduction in the protein digestibility of *Eulepida mashona* adults (beetle) and *Henicus whellani* adults (cricket) was also demonstrated after cooking, with cooking duration having no significant impact on digestibility. However, different results were obtained for frying. Although the protein digestibility of *Henicus whellani* decreased with frying, it had no statistically significant effect on the resulting protein digestibility of *Eulepida mashona* (17).

In our study, drying was the most suitable preparation method, leading to an almost 10% increase in digestibility. The favourable effect of drying on the digestibility of both species can be explained by the biochemical reactions occurring in foods during prolonged drying at relatively low temperatures (in our case at 80°C for 15 h). During drying, there was an increase in the fraction of free amino acids. These free amino acids contribute to the increased digestibility (26).

However, it is important to note that the way of killing by blanching, can itself influence the digestibility of the protein, as blanching may promote the unfolding of the polypeptide chain, making the protein more accessible to digestive enzymes. This method of killing edible insects could thus slightly increase the digestibility across all tested samples. Therefore, it would be appropriate to also focus on the impact of the killing method on protein digestibility in future research.

Because the overall determination of protein digestibility only considers the absorbed percentage of proteins, digestibility was also determined in the form of DIAAS in this study as it provides a more comprehensive view of protein quality. The DIAAS focuses on the need for essential amino acids (33). DIAAS values are divided into three main categories and numerically express the quality of the source in terms of essential amino acid content. The overall value, which allows the comparison of individual sources, is related to the amount of the limiting amino acid, that is, the essential amino acid that is least abundant in the protein. From this perspective, DIAAS values can be divided into three categories: 100% or more indicating excellent protein quality, between 75 and 99% indicating good protein quality, and less than 75%, for which it is not possible to make nutritional claims regarding protein quality (34, 35).

Similar to overall digestibility, *T. molitor* achieved higher DIAAS values than *G. assimilis* by roughly 15%. Hence, DIAAS is significantly influenced by the insect species. This finding was also confirmed in a study by Malla et al. (36), where DIAAS values ranged from 64 to 92% depending on the insect species.

In our experiment, we found that all culinary treatments positively influenced DIAAS. Moreover, drying was the most suitable treatment for both samples. Using the calculation based on crude protein (TN × 6.25), it was possible to classify all samples into the category of good protein sources, with *T. molitor* treated by drying classified as an excellent protein source. In our experiment, the DIAAS of *T. molitor* ranged from 95.72% to 105.44%, which is consistent with the values reported by Hammer et al. (35), who also determined DIAAS values above 90%.

Because the DIAAS calculation includes the conversion of the content of individual essential amino acids to the protein content in the sample, the overall result may be influenced by the accuracy of determining the protein content. Currently, the Kjeldahl method, which is based on determining the total nitrogen content in the analysed material, is most commonly used to evaluate protein content. The nitrogen content is then converted using a general conversion factor of 6.25, which allows for the estimation of the total protein content in the sample. However, this method can lead to an overestimation of protein content owing to the high chitin content in insect exoskeletons, leading to distortions between the calculated value and the real digestible protein content. For this reason, Boulos et al. (37) proposed a reduced conversion factor of 5.33 specifically for insects. However, since the general conversion factor of 6.25 is still commonly used in the literature for calculating protein content, we did not employ the reduced factor for insects in our calculations, but instead used the universal conversion factor of 6.25. The obtained results were then compared with those based on calculations relying on the determination of protein content as the sum of all amino acids. This conversion should lead to results that are unaffected by the possible overestimation of total protein content, thus enabling a more reliable assessment of protein quality. As expected, the two methods yielded different results. Using the calculation method based on crude protein (TN × 6.25), the DIAAS values for *T. molitor* ranged from 95.72% to 105.44% and for *G. assimilis* from 81.49% to 88.66%. When calculated based on the sum of anhydrous amino acids, all culinary treatments for both insect species resulted in DIAAS values higher than 100%. Thus, the use of the second calculation method led to an increase in DIAAS’s of approximately 20% for both insect species and all culinary treatments. An increase in DIAAS owing to different calculation methods was also observed in a previous study (35). However, it should be noted that using the reduced conversion factor of 5.33 for insects as reported by Boulos et al. (37), the obtained DIAAS values ranged from 112.24% to 123.64% for *T. molitor* and from 95.55% to 103.96% for *G. assimilis*. These values were much closer to those obtained using the calculation based on the sum of anhydrous amino acids.

Therefore, it is apparent that the choice of the DIAAS calculation method has a significant impact on the evaluation of protein digestibility in insects. By including the sum of anhydrous amino acids, we were able to eliminate the possible overestimation of the total protein content. Thus, we achieved a more reliable and objective assessment of protein quality in the examined insect species. This suggests that reassessing DIAAS calculations based on the anhydrous amino acid content significantly influences the results and contributes to a better understanding of the biological availability of essential amino acids in insect proteins.

## 5 Conclusion

We found a significant effect of culinary treatments on the digestibility and protein quality of the examined edible insect species, *T. molitor* and *G. assimilis*. Overall, protein digestibility was influenced by both insect species and culinary treatment methods. Drying was the most effective method leading to a significant increase in the digestibility of both insect species.

Moreover, the DIAAS calculation method significantly influenced the assessment of insect protein quality. Including the sum of the anhydrous amino acids eliminates the possible overestimation of the total protein content and contributes to a more objective assessment of the biological availability of essential amino acids in insect proteins. This methodology could be a crucial step towards more accurately determining the suitability of insects as a protein food source for human nutrition.

The study brings the new information about the protein digestibility after selected culinary treatments, which could be in the interest of, e.g., nutrition specialists. Additionally, the study discusses the DIAAS calculation method, which involves determining the sum of amino acids and provides more realistic results.

## Data availability statement

The raw data supporting the conclusions of this article will be made available by the authors, without undue reservation.

## Author contributions

BL: Conceptualization, Formal analysis, Investigation, Methodology, Validation, Writing – original draft, Writing – review & editing, Data curation. ID: Conceptualization, Formal analysis, Writing – review & editing, Project administration, Writing – original draft. MaK: Methodology, Writing – review & editing. MiK: Formal analysis, Writing – review & editing. LK: Conceptualization, Resources, Supervision, Writing – review & editing.
