# Supplementary material for: Culinary treatments impact the digestibility and protein quality of edible insects: a case study with Tenebrio molitor and Gryllus assimilis
Source: Front Nutr. 2024 May 31;11:1399827. doi: 10.3389/fnut.2024.1399827 (PMC11179427; doi:10.3389/fnut.2024.1399827)
Supplement: Supplementary file 1 [file Table_1.docx]

Supplementary Material

Table S1: DIAA and DIAAS of *Tenebrio* *molitor* and *Gryllus* *assimilis* calculated using two methods. The first method involved recalculating the DIAAs to 1 g of crude protein, obtained by multiplying the total nitrogen by a conversion factor of 6.25 (TN × 6.25). The second method utilised the sum of individually determined amino acids in dry matter (the sum of anhydrous AA).

| **DIAA a DIAAS (TN x 6.25)** | | | | | | | | | | |
| --- | --- | --- | --- | --- | --- | --- | --- | --- | --- | --- |
| Insect | *Tenebrio molitor* | | | | | *Gryllus assimilis* | | | | |
| Culinary treatment | No treatment | Boiled | Roasted | Dried | Microwaved | No treatment | Boiled | Roasted | Dried | Microwaved |
| Histidine | 1.61 ± 0.11^ab^ | 1.68 ± 0.12^ab^ | 1.61 ± 0.11^ab^ | 1.63 ± 0.11^ab^ | 1.72 ± 0.12^a^ | 1.38 ± 0.10^ab^ | 1.33 ± 0.09^ab^ | 1.37 ± 0.10^ab^ | 1.30 ± 0.09^b^ | 1.31 ± 0.09^b^ |
| Isoleucine | 1.39 ± 0.10^a^ | 1.45 ± 0.10^a^ | 1.49 ± 0.10^a^ | 1.56 ± 0.11^a^ | 1.41 ± 0.10^a^ | 1.31 ± 0.09^a^ | 1.23 ± 0.09^a^ | 1.39 ± 0.10^a^ | 1.38 ± 0.10^a^ | 1.29 ± 0.09^a^ |
| Leucine | 1.08 ± 0.08^a^ | 1.13 ± 0.08^a^ | 1.15 ± 0.08^a^ | 1.19 ± 0.08^a^ | 1.15 ± 0.08^a^ | 1.06 ± 0.07^a^ | 1.03 ± 0.07^a^ | 1.09 ± 0.08^a^ | 1.08 ± 0.08^a^ | 1.05 ± 0.07^a^ |
| Lysine | 0.96 ± 0.07^a^ | 0.98 ± 0.07^a^ | 0.99 ± 0.07^a^ | 1.05 ± 0.07^a^ | 0.99 ± 0.07^a^ | 0.81 ± 0.06^a^ | 0.83 ± 0.06^a^ | 0.83 ± 0.06^a^ | 0.89 ± 0.06^a^ | 0.82 ± 0.06^a^ |
| SAA | 1.22 ± 0.09^ab^ | 1.21 ± 0.08^ab^ | 1.29 ± 0.09^ab^ | 1.41 ± 0.10^a^ | 1.23 ± 0.09^ab^ | 1.06 ± 0.07^b^ | 1.06 ± 0.07^b^ | 1.14 ± 0.08^ab^ | 1.11 ± 0.08^ab^ | 1.16 ± 0.08^ab^ |
| AAA | 2.90 ± 0.20^ab^ | 3.06 ± 0.21^a^ | 2.87 ± 0.20^abc^ | 2.81 ± 0.20^abcd^ | 2.90 ± 0.20^ab^ | 2.04 ± 0.14^e^ | 2.20 ± 0.15^cde^ | 2.18 ± 0.15^de^ | 2.12 ± 0.15^e^ | 2.36 ± 0.17^bcde^ |
| Threonine | 1.48 ± 0.10^a^ | 1.44 ± 0.10^a^ | 1.50 ± 0.10^a^ | 1.56 ± 0.11^a^ | 1.48 ± 0.10^a^ | 1.29 ± 0.09^a^ | 1.28 ± 0.09^a^ | 1.35 ± 0.09^a^ | 1.39 ± 0.10^a^ | 1.31 ± 0.09^a^ |
| Tryptophan | 2.08 ± 0.10^a^ | 2.02 ± 0.10^a^ | 2.08 ± 0.10^a^ | 2.15 ± 0.11^a^ | 2.07 ± 0.10^a^ | 1.87 ± 0.09^ab^ | 1.63 ± 0.08^b^ | 1.79 ± 0.09^ab^ | 1.83 ± 0.09^ab^ | 1.80 ± 0.09^ab^ |
| Valine | 1.31 ± 0.09^a^ | 1.38 ± 0.10^a^ | 1.33 ± 0.09^a^ | 1.37 ± 0.10^a^ | 1.34 ± 0.09^a^ | 1.37 ± 0.10^a^ | 1.30 ± 0.09^a^ | 1.43 ± 0.10^a^ | 1.44 ± 0.10^a^ | 1.35 ± 0.09^a^ |
| DIAAS (%) | 95.72 ± 6.70^a^ | 98.19 ± 6.87^a^ | 99.29 ± 6.94^a^ | 105.44 ± 7.37^a^ | 99.09 ± 6.94^a^ | 81.49 ± 6.20^a^ | 82.71 ± 5.55^a^ | 82.82 ± 5.80^a^ | 88.66 ± 5.78^a^ | 81.66 ± 5.97^a^ |
| **DIAA a DIAAS (sum of AA)** | | | | | | | | | | |
| Culinary treatment | No treatment | Boiled | Roasted | Dried | Microwaved | No treatment | Boiled | Roasted | Dried | Microwaved |
| Histidine | 1.94 ± 0.14^a^ | 2.01 ± 0.14^a^ | 1.98 ± 0.14^a^ | 1.91 ± 0.13^a^ | 2.07 ± 0.14^a^ | 1.76 ± 0.12^a^ | 1.70 ± 0.12^a^ | 1.73 ± 0.12^a^ | 1.61 ± 0.11^a^ | 1.69 ± 0.12^a^ |
| Isoleucine | 1.68 ± 0.12^a^ | 1.73 ± 0.12^a^ | 1.84 ± 0.13^a^ | 1.83 ± 0.13^a^ | 1.69 ± 0.12^a^ | 1.68 ± 0.12^a^ | 1.58 ± 0.11^a^ | 1.76 ± 0.12^a^ | 1.69 ± 0.12^a^ | 1.66 ± 0.12^a^ |
| Leucine | 1.30 ± 0.09^a^ | 1.34 ± 0.09^a^ | 1.41 ± 0.10^a^ | 1.40 ± 0.10^a^ | 1.38 ± 0.10^a^ | 1.35 ± 0.09^a^ | 1.32 ± 0.09^a^ | 1.38 ± 0.10^a^ | 1.33 ± 0.09^a^ | 1.35 ± 0.09^a^ |
| Lysine | 1.15 ± 0.08^a^ | 1.17 ± 0.08^a^ | 1.22 ± 0.09^a^ | 1.24 ± 0.09^a^ | 1.19 ± 0.08^a^ | 1.04 ± 0.08^a^ | 1.06 ± 0.07^a^ | 1.04 ± 0.07^a^ | 1.09 ± 0.07^a^ | 1.05 ± 0.08^a^ |
| SAA | 1.47 ± 0.10^a^ | 1.44 ± 0.10^a^ | 1.59 ± 0.11^a^ | 1.66 ± 0.12^a^ | 1.48 ± 0.10^a^ | 1.36 ± 0.10^a^ | 1.36 ± 0.10^a^ | 1.43 ± 0.10^a^ | 1.37 ± 0.10^a^ | 1.49 ± 0.10^a^ |
| AAA | 3.50 ± 0.25^ab^ | 3.66 ± 0.26^a^ | 3.53 ± 0.25^ab^ | 3.30 ± 0.23^abc^ | 3.48 ± 0.24^ab^ | 2.60 ± 0.18^c^ | 2.82 ± 0.20^bc^ | 2.75 ± 0.19^bc^ | 2.61 ± 0.18^c^ | 3.04 ± 0.21^abc^ |
| Threonine | 1.78 ± 0.12^a^ | 1.72 ± 0.12^a^ | 1.84 ± 0.13^a^ | 1.84 ± 0.13^a^ | 1.77 ± 0.12^a^ | 1.65 ± 0.12^a^ | 1.65 ± 0.12^a^ | 1.70 ± 0.12^a^ | 1.71 ± 0.12^a^ | 1.68 ± 0.12^a^ |
| Tryptophan | 2.51 ± 0.13^a^ | 2.42 ± 0.12^a^ | 2.56 ± 0.13^a^ | 2.53 ± 0.13^a^ | 2.48 ± 0.12^a^ | 2.39 ± 0.12^a^ | 2.09 ± 0.10^b^ | 2.26 ± 0.11^a^ | 2.25 ± 0.11^a^ | 2.32 ± 0.12^a^ |
| Valine | 1.58 ± 0.11^a^ | 1.65 ± 0.12^a^ | 1.64 ± 0.11^a^ | 1.61 ± 0.11^a^ | 1.60 ± 0.11^a^ | 1.74 ± 0.12^a^ | 1.67 ± 0.12^a^ | 1.80 ± 0.13^a^ | 1.77 ± 0.12^a^ | 1.74 ± 0.12^a^ |
| DIAAS (%) | 115.44 ± 8.08^a^ | 117.30 ± 8.21^a^ | 122.14 ± 8.54^a^ | 123.86 ± 8.66^a^ | 118.79 ± 8.32^a^ | 103.99 ± 7.92^a^ | 106.11 ± 7.13^a^ | 104.43 ± 7.32^a^ | 109.17 ± 7.12^a^ | 105.20 ± 7.69^a^ |

DIAA, digestible indispensable amino acid reference ratio; DIAAS, digestible indispensable amino acid score; SAA: sulphur amino acid; AAA, aromatic amino acid; AA, amino acids; TN, total nitrogen. Values are expressed as means ± standard deviations (n = 3), numbers on the same row followed by different lowercase letters are statistically different (p ≤ 0.05).
